# Effect of cdp-choline on microcirculatory alterations during endotoxemia

**DOI:** 10.1186/cc11941

**Published:** 2013-03-19

**Authors:** K Schmidt, M Doerr, T Brenner, S Hofer, A Walther

**Affiliations:** 1 Universitätsklinikum Heidelberg, Germany; 2 Klinikum Stuttgart, Klinik für Anästhesiologie u. operative Intensivmedizin, Stuttgart, Germany

## Introduction

The cholinergic anti-inflammatory pathway (CAP) is a physiological mechanism that inhibits cytokine production and minimizes tissue injury during inflammation. CAP-mediated anti-inflammatory signals in vagal efferent nerve fibers result in the release of acetylcholine, which interacts with innate immune cells that express the nicotinic acetylcholine receptor subunit α7 (α7nAChR).

Endothelial dysfunction during sepsis is responsible for increased endothelial permeability, leukocyte-endothelial interaction and functional breakdown of microvascular perfusion. Endotoxemia-induced endothelial dysfunction can be reduced by cholinergic CAP activation [1]. The aim of this study was to determine the effects of the α7nAChR-agonist cdp-choline on microcirculatory alterations during experimental endotoxemia.

## Methods

Using fluorescent intravital microscopy, we determined venular wall shear rate, macromolecular efflux and leukocyte adhesion in mesenteric postcapillary venules of male Wistar rats. Endotoxemia was induced over 120 minutes by intravenous infusion of lipopolysaccharide (LPS). Control groups received an equivalent volume of saline. Cdp-choline was applied as an i.v. bolus in treatment groups. Animals received either (i) saline alone, (ii) cdp-choline 10 minutes prior to saline administration, (iii) cdp-choline 10 minutes prior to LPS administration, (iv) cdp-choline 30 minutes after LPS administration or (v) LPS alone.

## Results

There were no significant differences in venular wall shear rate between the groups after 120 minutes. There was no significant difference in the number of adhering leukocytes between the cdp-choline/LPS groups (iii, iv) and the LPS group after 120 minutes. Macromolecular efflux significantly increased in all groups over 120 minutes. All groups (i, ii, iii, iv) showed a significantly reduced macromolecular efflux compared with the LPS group after 120 minutes.

## Conclusion

Cdp-choline has no effect on leukocyte-endothelial interaction and microhemodynamic alterations during endotoxemia. By activating the CAP, cdp-choline reduces capillary leakage. Thus cdp-choline might have a prophylactic and therapeutic anti-inflammatory effect on LPS-induced endothelial permeability. These findings identify the endothelium as a target of anti-inflammatory cholinergic mediators and cdp-choline as a potential therapeutic substance in sepsis treatment.
